# A new synonym for *Zelia* Robineau-Desvoidy, 1830 (Diptera, Tachinidae), the genus Opsozelia Townsend, 1919, with the description of three new species

**DOI:** 10.3897/zookeys.880.35482

**Published:** 2019-10-14

**Authors:** Rodrigo de Vilhena Perez Dios, Marcelo Domingos de Santis

**Affiliations:** 1 Department of Zoology, Institute of Biosciences, University of São Paulo, Rua do Matão, Travessa 14, n.101, Cidade Universitária, São Paulo-SP, 05508-900, Brazil University of São Paulo São Paulo Brazil

**Keywords:** description, Dexiinae, parasitoid, redescription, taxonomy

## Abstract

The monotypic tachinid genus *Opsozelia* Townsend, 1919 (Diptera: Tachinidae) is synonymized with *Zelia*Robineau-Desvoidy 1830, **syn. nov**. The single species of *Opsozelia*, *O. discalis* Townsend, 1919, is redescribed as *Zelia discalis*, **comb. nov.**, based on examination of the holotype from Guyana and additional material from Suriname, Brazil and Paraguay. Three new species of *Zelia* similar to *Z. discalis* are described from Brazil: *Z. magna***sp. nov.**, *Z. guimaraesi***sp. nov.** and *Z. formosa***sp. nov**. These four species are treated informally as the *Zelia discalis* species group. An identification key to the species of this species group is provided based on male specimens. Descriptions and illustrations are provided for the male terminalia of all species and for the female terminalia of one species, *Z. guimaraesi*.

## Introduction

*Zelia* is a New World genus that was composed, prior to this study, of 19 species (Guimarães 1971, O’Hara and Wood 2004). It was erected by Robineau-Desvoidy (1830) for five species, including *Zelia rostrata* Robineau-Desvoidy, 1830 (= *Dexia vertebrata* Say, 1829) from North America, designated as type species by Coquillett (1910). Of the other four species, three were left unplaced in the Tachinidae by O’Hara and Wood (2004) (*Z. analis* Robineau-Desvoidy, 1830, *Z. apicalis* Robineau-Desvoidy, 1830, and *Z. veloz* Robineau-Desvoidy, 1830) and the fourth was placed in *Ptilodexia* Brauer & Bergenstamm, 1889 by Guimarães (1971) (*Zelia strenua* Robineau-Desvoidy, 1830).

*Zelia* was enlarged by subsequent generic synonymies, as follows: Townsend (1919) synonymized *Leptoda* van der Wulp, 1885 with *Zelia*; Aldrich (1929) provided new descriptions for most of the Neotropical species and synonymized *Melaleuca* Wulp, 1891 and *Euzelia* Townsend, 1915 with *Zelia*; and Wood (1987) synonymized *Metadexia* Coquillett, 1899 and *Minthozelia* Townsend, 1919 with *Zelia* (as reviewed by O’Hara and Wood 1998). Earlier, Reinhard (1946) revised a portion of *Zelia*, as *Minthozelia*, including six new species. *Zelia* currently comprises 12 Nearctic and seven Neotropical species. However, the Neotropical species lack a key and are poorly known, being just referenced by lists and catalogues after the original descriptions.

The genus *Opsozelia* was described in the tribe Zeliini by Townsend (1919) for his new species, *O. discalis*, based on a single male from Bartica, Guyana. Later, Townsend (1931a) discovered that his new species was a synonym of *Musca lateralis* Fabricius, 1805, and changed the species name to *Opsozelia lateralis* (Fabricius, 1805). In his catalogue of Neotropical Tachinidae, Guimarães (1971) followed Townsend (1931a) in recognizing *Opsozelia* as a valid genus with the single species, *O. lateralis* (with *discalis* in synonymy). Thompson and Pont (1994) noted that the specific name of Fabricius was preoccupied by *Musca lateralis* Linnaeus, 1758 and the valid name of the species then reverted to Townsend’s original one, *Opsozelia discalis*.

The Zeliini were recognized as a tribe of 11 genera in the Americas south of the United States by Guimarães (1971) and a twelfth genus (*Neozelia*) was later described by Guimarães (1975). Tschorsnig (1985) examined one zeliine genus, *Diaugia* Perty, 1833, and placed it in the Dexiini based on male terminalic features. Ziegler (1998) studied the larval cephaloskeleton of third instar larvae, and puparia, of some *Zelia* species and did not find any discernable differences from the Dexiini. O’Hara and Wood (2004) did not recognize Zeliini and incorporated the only North American genus, the type genus *Zelia*, within a more broadly defined Dexiini. This action was recently supported in the recent molecular study of Stireman et al. (2019) in which *Zelia* was nested within Dexiini. We follow the foregoing authors in classifying the Zeliini*sensu*Guimarães (1971) within the Dexiini but note that these *Zelia*-group genera may form a monophyletic clade within Dexiini and some of them (in addition to *Opsozelia*, treated herein) may prove to be congeneric with *Zelia*.

In the present paper, the monotypic genus *Opsozelia* is synonymized with *Zelia*. We also present a small diagnosis for Dexiini and some morphological characters that help identify *Zelia*. We recognize and characterize a group of species within *Zelia* that have at least two discal setae on abdominal tergites III and IV and call this group the *Zelia discalis* species group. This species group is revised and four Neotropical species are recognized: *Z. discalis* (Townsend) from Guyana and three new species from Brazil, namely *Z. formosa* sp. nov., *Z. guimaraesi* sp. nov. and *Z. magna* sp. nov. The new species are described, *Z. discalis* is redescribed, and an identification key to the males of this species group is provided. Descriptions and illustrations of the male terminalia of all species are presented for the first time. The female of *Z. guimaraesi* sp. nov. is described and illustrated.

## Materials and methods

The examined material was deposited at the following institutions: Museu de Zoologia, Universidade de São Paulo, São Paulo, Brazil (**MZSP**), Museu Nacional do Rio de Janeiro, Rio de Janeiro, Brazil (**MNRJ**) and National Museum of Natural History, Smithsonian Institution, Washington, D.C., USA (**USNM**). The labels of type material are represented with quotation marks (“) to indicate the same label, a slash (/) for line break, and a semicolon to indicate a new label.

Male terminalia were cleared in a 10% solution of KOH at room temperature for ca. 24 hours, then neutralized with acetic acid (10%) and washed with distilled water and a series of ethanol solutions at increasing concentrations. After examination, the terminalia were placed in plastic microvials filled with glycerin and pinned with their respective specimens. All specimens were measured with the software LEICA LAS version 4.1.0. Photographs were taken using a Leica DFC420 digital camera attached to a Leica MZ16 stereomicroscope, and the software LEICA LAS version 4.1.0. The images were subsequently stacked using Helicon Focus 5.3 and edited in Adobe Photoshop CS6. Illustrations were made using a Leica MZ16 stereomicroscope with camera lucida attached and edited in Adobe Illustrator CS6. Morphological terminology follows Cumming and Wood (2017).

## Taxonomic part

### Genus Zelia

Taxon classificationAnimaliaDipteraTachinidae

Robineau-Desvoidy, 1830

9CB827D1-A226-5895-B69C-78F765E7E59E

Zelia Robineau-Desvoidy, 1830: 314. Type species: Zelia rostrata Robineau-Desvoidy, 1830 (= Dexia vertebrata Say, 1829), by subsequent designation of Coquillett, 1910: 621.
Leptoda van der Wulp, 1885: 196. Type species: Dexia gracilis Wiedemann, 1830 (= Dexia vertebrata Say, 1829), by subsequent designation of van der Wulp, 1891: 250.
Melaleuca van der Wulp, 1891: 213. Type species: Melaleuca spectabilis van der Wulp, by subsequent monotypy of van der Wulp, 1891: 247.
Metadexia Coquillett, 1899: 220. Type species: Metadexia tricolor Coquillett, 1899, by monotypy.
Euzelia Townsend, 1915: 23. Type species: Zelia wildermuthii Walton, 1914, by original designation.
Minthozelia Townsend, 1919: 556. Type species: Minthozelia montana Townsend, 1919, by original designation.
Opsozelia Townsend, 1919: 557. Type species: Opsozelia discalis Townsend, 1919 (= Musca lateralis Fabricius, 1805), by original designation, **syn. nov.**

#### Notes.

The genus *Zelia* is a group with 19 valid species at present. This study began as a revision of the previously valid genus *Opsozelia*, but later it was recognized as a synonym of *Zelia.* Since the original intent of this study was not to revise *Zelia*, we were not able to examine all *Zelia* species. Therefore, we cannot provide a full description for the genus. Instead, we provide a small diagnosis for the genus, as well as some features for the Dexiini, modifying what was stated by Thompson (1963) and Mesnil (1980). The species of *Zelia* with at least two discal setae on abdominal tergites III and IV (i.e., members of the *Z. discalis* species group), are then revised.

#### Diagnosis.

*Zelia* shares with Dexiini the following characters that, simultaneously, differs from other Tachinidae: compound eye bare (except for *Callotroxis* Aldrich, 1929 and *Huascarodexia* Townsend, 1919). Front narrow and without orbital setae in male, broad and with one proclinate orbital seta in female. Lunula bare. Frontal setae, forwardly directed or crossed, anteriorly reaching external angle of lunula, or slightly in front of it, but never descending to parafacial (except in *Psecacera* Bigot, 1880, *Morphodexia* Townsend, 1931 and *Dasyuromyia* Bigot, 1885). Facial carina absent. Antenna short, inserted at level or below half of compound eye height, thickened only at its base, often pubescent or plumose. Thorax with scutellum with decussate (rarely parallel) apical setae, normally without lateral setae. Abdominal sternites usually completely covered by ventrolateral margins of corresponding tergites.

*Zelia* differs from other Dexiini genera by the following combination of characters: Head silver pruinose (golden pruinose in *Myiomima* Brauer & Bergenstamm, 1889). Pedicel with one long seta and various setulae on its surface (in *Neozelia* Guimarães, 1975 with a tuft of long setulae). Postpedicel long, compressed laterally. Arista long plumose (bare in *Psecacera*). Facial carina absent (e.g., present in *Platyrrhinodexia* Townsend, 1927). Haustellum short, ca. 0.5× the head height (e.g., two or three times in *Prosenoides* Brauer & Bergenstamm, 1891). Thorax with proepisternum and prosternum bare (e.g., setulose in *Tromodesiana* Townsend, 1931). Intrapostalar seta absent. Scutellum with just regular setae (e.g., various upturned setae in *Tropidopsiomorpha* Townsend, 1927). Wing hyaline (smoky in in *Yahuarmayoia* Townsend, 1927; with maculae in *Scotiptera* Macquart, 1835). Costal spine undeveloped. Abdomen strongly pointed apically, especially in male (in *Yahuarmayoia* and *Z. discalis* species group is broad, excluding *Z. formosa* sp. nov.). Male abdomen somewhat elongate (not elongate in *Ophirodexia* Townsend, 1911). Abdominal tergites with just one row of setae (e.g., in *Hystrichodexia* Röder, 1886, two or three rows of discal setae). Tergite IV with three to five discals or without discals (e.g., with discal setae in sytergite I+II to V in *Ptilodexia*).

Justification for the synonymy of *Opsozelia* with *Zelia* and the *Z. discalis* group of species with at least two discal setae on tergites III and IV:

All of the examined species of the *Z. discalis* species group are similar to *Zelia* species, including the terminalia. These species do not present any outstanding morphological features that justify a separate genus. However, considering the difficulty in identifying the Neotropical Dexiini and *Zelia* species, we keep maintain species in their own species group for identification purposes. These species are easily recognized among other *Zelia* by the presence of at least two discal setae on abdominal tergites III and IV (other *Zelia* species without discal setae on these tergites).

#### Description of the *Zelia discalis* species group

Male holoptic and female dichoptic. Compound eye bare. Frontal vitta and ocellar triangle dark brown. Head light yellow to tawny, covered entirely with silver pruinosity. Minute proclinate setae on fronto-orbital plate. Parafacial bare. Ocellar setae proclinate and well differentiated from the adjacent setae; postocellar setae proclinate. Inner and outer vertical setae subparallel and convergent. No facial carina. Genal dilation with pale pruinosity and covered with black setulae. Facial ridge with small setulae near vibrissal insertion. Antenna inserted below middle of compound eye. Arista densely plumose. Strong and convergent vibrissae; four or five developed subvibrissal setae. Palpus cylindrical and a little clavate. Thorax brown to dark brown with silver or light golden pruinosity. Prescutum with four dark vittae, the two inner vittae thinner than the outer vittae. Prosternum and proepisternum bare. Notopleuron with two equal-sized setae. Two proepimeral setae. Two proepisternal setae. Three katepisternal setae, the lower one weaker. Postalar callus with two large and one smaller setae. Anepimeron with a single long seta. Anatergite bare. Katepimeron with setulae anteriorly. Costal spine absent. Vein M1 ending at wing margin close to tip. Abdomen conical, basally large and rounded, tapering to tip. Mid-dorsal depression on syntergite I+II reaching the posterior margin. Syntergite I+II and tergite III with one pair of median marginal setae. Tergite III and IV with 2–4 pairs of discal setae. Tergite IV with one row of median marginal setae and approximately ten discal seta decreasing in size anteriorly. Tergite V with one row each of marginal and discal setae. Sternites hidden. Male terminalia with cerci separated and pointed, larger basally. Surstylus broad, and usually rounded at tip, sometimes slightly pointed. Pregonite and postgonite fused as curved elongate structure, without a distinct separation; pregonite connected basally to the hypandrium by a membrane (sometimes thin, almost sclerotized). Epiphallus present, fused with basiphallus. Basiphallus varying in size. Distiphallus with extension of dorsal sclerite varying in size; dorsal sclerite ventrally serrulated; granular zone present, varying in size.

### Key to species of *Zelia discalis* species group (males).

**Table:** 

1	Lower margin of face protruding below vibrissal angle; width of fronto-orbital plate 0.5× or less the height of gena	**2**
–	Lower margin of face not protruding below vibrissal angle; width of fronto-orbital plate 0.8× or more the height of gena	**3**
2	Scutum with 4+3 acrostichal setae. Scutellum with two pairs of discal setae. Abdomen pale yellow, with median brown rounded vitta covering syntergite I+II, tergite III with small brownish black spot, at the insertion of the marginal median seta; posterior margin of tergite IV with a triangular spot, covering approx. the posterior ¼; tergite V entirely brownish black, without pruinosity	***Zelia magna* sp. nov.**
–	Scutum with 2+2 acrostichal setae. Scutellum with a single pair of discal setae. Abdomen pale yellow, with median brown longitudinal vitta covering syntergite I+II, continuing along the middle of the abdomen and ending at the middle of tergite V; posterior margin of tergite IV reddish brown; posterolateral margins of syntergite I+II and tergites III and IV with a brown spot; tergite V, reddish brown with silver pruinosity laterally	***Zelia discalis* (Townsend, 1919), comb. nov.**
3	Thorax with postscutum with pale pruinosity only on the anterior region, not forming vittae; wing dorsally with vein R_4+5_ setulose for 1/4 of distance to crossvein dm-cu; abdominal syntergite I+II with one pair of median marginal setae; syntergite I+II to tergite IV pale yellow, each tergite with a brownish black vitta medially, broadening posteriorly (3× the width of the anterior portion) and with white pruinosity laterally	***Zelia formosa* sp. nov.**
–	Thorax with postscutum brown to dark brown with 4 vittae of silver pruinosity; the inner vittae half the length of the outer ones, neither reaching the scutellum; wing dorsally with setulae only on base of R_4+5_; abdominal syntergite I+II without median marginal setae; abdomen pale yellow, with median brown longitudinal vitta covering syntergite I+II, continuing along the middle of the abdomen to the end of tergite IV; tergite V entirely reddish black with silver pruinosity laterally	***Zelia guimaraesi* sp. nov.**

### Zelia discalis

Taxon classificationAnimaliaDipteraTachinidae

(Townsend, 1919)

E8BC6A89-2CBC-586C-A01E-57A8CF909A23

Figs 1 , 7 A–C

Opsozelia discalis Townsend, 1919: 557. Holotype ♂ (USNM; examined). Type locality: Guyana, Bartica, Kartabo.

#### Type material examined.

Holotype ♂: “Bartica, BG / VII.10.1901” “Type No. / 22237 / U.S.N.M.” “Opsozelia / discalis / ♂ T. Det CHTT”.

#### Additional material examined.

Suriname: [unreadable label], 1 ♂, 4.xi.1942 (MZSP); Brasil: *Pará*, Oriximiná, Rio Cumina, Cachoeira da Paciência, 1 ♂, 8.x.1936, Almeida col. (MZSP); *São Paulo*, São Paulo, Alto da Serra, 1 ♂, ii.1926 R. Spitz col. (MZSP); Cantareira, Horto Florestal, 1 ♂, L. Travassos col. (MZSP); *Santa Catarina*, Nova Teutônia, 27 ♂, i.1966, 8 ♂, ii.1966, 3 ♂, iv.1966, 2 ♂, x.1966, 1 ♂, xii.1970, F. Plaumann col. (MZSP); Paraguay: Amambay, Caballera, 1 ♂, 23–25.xi.1971 (MZSP).

#### Diagnosis.

Frontal vitta width, in the narrowest point, narrower than ocellar triangle; postpedicel entirely yellow to orange; halter entirely pale yellow-tawny.

#### Description.

***Body length***: 12.0 mm.

***Coloration***: Occiput with long and pale setulae. Antenna yellow to orange. Palpus yellow-orange. Thorax with postscutum with four dark vittae, the inner vittae half the length of the outer, neither reaching the scutellum. Scutellum dark brown, with pale pruinosity posteriorly. Subscutellum with pale pruinosity. Wing hyaline, slightly light brown along the veins. Calypteres white-pale translucent. Halter pale yellow-tawny. Posterior spiracle light brown. Legs brown to tawny with silver pruinosity on coxae and femora; tarsi darker. Claws brown with tip darker. Abdomen pale yellow, with median brown longitudinal vitta covering syntergite I+II, continuing along the middle of the abdomen and ending at the middle of tergite V; posterior margin of tergite IV and tergite V reddish brown; posterolateral margins of tergites III, IV, and V with a brown spot.

**Figure 1.**
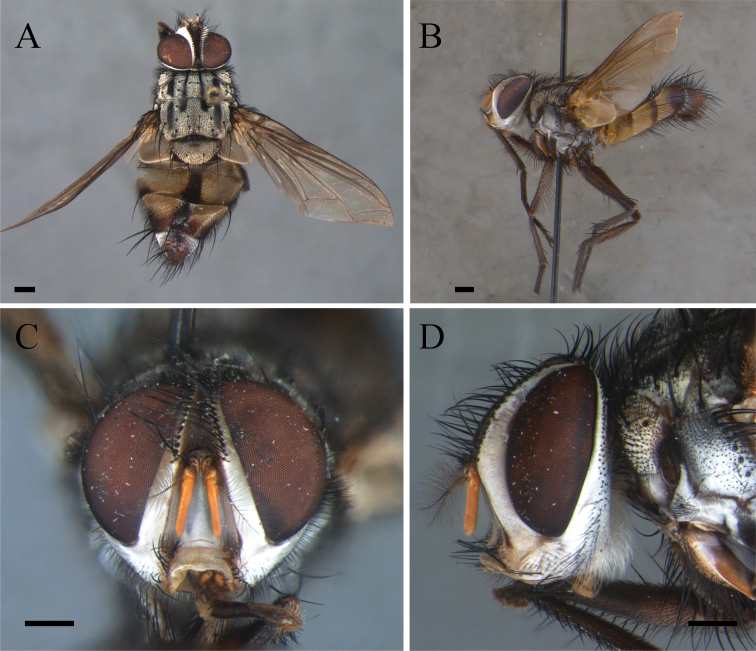
*Zelia discalis*, holotype, male. **A** Dorsal habitus **B** lateral habitus **C** head frontal **D** head lateral. Scale bars: 1 mm.

***Head***: Frontal vitta at its widest point approx. as wide as the vertex in dorsal view. Frontal vitta, in the narrowest point, narrower than ocellar triangle. Fronto-orbital plate 17 pairs of proclinate setae minute proclinate setae; narrower than frontal-vitta and parafacial. Ocellar setae proclinate divergent. Orbital plate with three setulae. Postpedicel slender, 3× the combined length of scape and pedicel; arista plumose with two or three dorsal and one ventral rows; length of longest cilia ca. 7× basal width of arista. Facial ridge with ten or eleven setulae on lower third. Lower facial margin protruding, and visible in profile. Vibrissa long, inserted above lower facial margin. Prementum as long as palpus. Labella developed, little longer than 0.5× the prementum.

***Thorax***: Acrostichals 2+2. Dorsocentral 3+3. Intra-alar 1+2, first post-sutural weak; intra-postalar absent. Supra-alar 2+3, first post-sutural weak. Postpronotal lobe with four setae, three forming an anterior row and one posterior. Anepisternum with eight strong setae and two upwardly directed setulae anteriorly. Scutellum with one basal, one lateral, one weak subapical, one apical and one discal pairs of setae.

***Wing***: Base of R dorsally and ventrally setulose. M vein bent forward to R_4+5_, forming an angle slightly smaller than 90°, and convex after bend.

***Legs***: Fore coxa with many setae anteriorly; fore femur with dorsal and posteroventral rows of setae; fore tibia with two posterior setae and row of shorter anterodorsal setae. Mid femur with three posteroventral basal setae, three dorsal to posterodorsal preapical setae; mid tibia with one anteroventral median seta, two anterior median setae and two posterior median setae. Hind femur with three anteroventral setae on basal half and three ventral setae on basal half and with row of anterodorsal setae; one posterodorsal preapical setae; hind tibia with two anterior median, two anteroventral median and two posterodorsal median setae. Claws straight with the tip curved.

***Abdomen***: Syntergite I+II without pair of median margin setae. Tergite III with three discal setae decreasing in size anteriorly one median marginal seta and one lateral marginal seta. Tergite IV with four discal setae decreasing in size anteriorly a marginal row of median lateral. Ventral borders of tergites with a row of median setae.

***Terminalia*** (Fig. 7A–C): Tergite VI brown and segment VII+VIII brownish black with silver pruinosity. Surstyili in lateral view narrow, less than 2× the maximum cerci width; triangular shaped, narrow at the apex. Surstyli in posterior view without an expansion laterally. Apex of the hypandrium directed backwards. Middle bar subequal to the total length of the granular structure of distiphallus. Apex of distiphallus not curved.

#### Notes on type.

Missing left vibrissa and right one broken in half. Missing right mid femur and so on; missing right trochanter and so on. Abdomen damaged, basally compressed and smashed, and with a rupture on the left side of tergite III.

#### Variation.

Orbital plate with 2–7 setulae. Intra-alar - 2+3. Supra-alar - 2+4.

#### Distribution.

Suriname, Brazil (Pará, São Paulo and Santa Catarina states), and Paraguay (Amambay department).

### Zelia magna

Taxon classificationAnimaliaDipteraTachinidae

Dios & de Santis sp. nov.

B11D0891-C2BD-506C-BFC2-6B1CA4ADE54B

http://zoobank.org/7E6A133A-FF41-430E-A2C0-BFA6A9991577

Figs 2 , 7 D–F

#### Type material examined.

***Holotype*** ♂: BRAZIL: *Rio de Janeiro*, Nova Friburgo, Mury, xii.1980, Gred & Guimarães leg. (MZSP). Labelled as follows: “Mury, Nova Friburgo / Rio de Janeiro – Br. / xii.1980 / Gred & Guimarães col.” [printed label]; “Zelia / magna sp. nov. / Dios & Santis det. 2016” [handwriting/printed label]; “Holotipo” [red label]. Paratypes: BRAZIL: *Rio de Janeiro*, Itatiaia (700 m), 1 ♂, 3.iv.1928, J.F. Zikán col. (MZSP); *São Paulo*, São Paulo, Horto Florestal, 1 ♂, 13.ii.1944, Ramalho col. (MZSP).

#### Diagnosis.

Thorax with scutellum with two pairs of discals; abdomen pale yellow, syntergite I+II with median brown rounded vitta; tergite III with small brownish black spot, at the insertion of the marginal median seta; tergite IV posterior margin with a triangular spot, covering approx. the posterior ¼; posterolateral margins of syntergite I+II and tergites III and IV with a brown spot; legs dark brown; light brown claws; tergite V, entirely brownish black, without pruinosity; tergite VI and segment VII+VIII brownish black; largest species of the genus.

#### Description.

***Body length***: 17.8 mm

***Coloration***: Occiput with long and pale setulae. Antenna yellowish grey-dusted. Palpus yellow-orange. Thorax with postscutum with four dark vittae, the inner vittae half the length of the outer, neither reaching the scutellum. Scutellum dark brown, with pale pruinosity posteriorly. Subscutellum with pale pruinosity. Wing hyaline, slightly light brown along the veins. Calypteres slightly infuscated. Halter and posterior spiracle light brown. Legs brown with silver pruinosity on coxae and femora; tarsi darker. Claws brown with tip darker. Abdomen pale yellow, with median brown longitudinal vitta covering syntergite I+II, in tergite III a brownish black dot at the insertion of the median marginals, in tergite IV a brownish black triangular spot on the posterior region and in V; and tergite V entirely brownish black without pruinosity; posterolateral margin of tergite IV with a brown spot.

**Figure 2.**
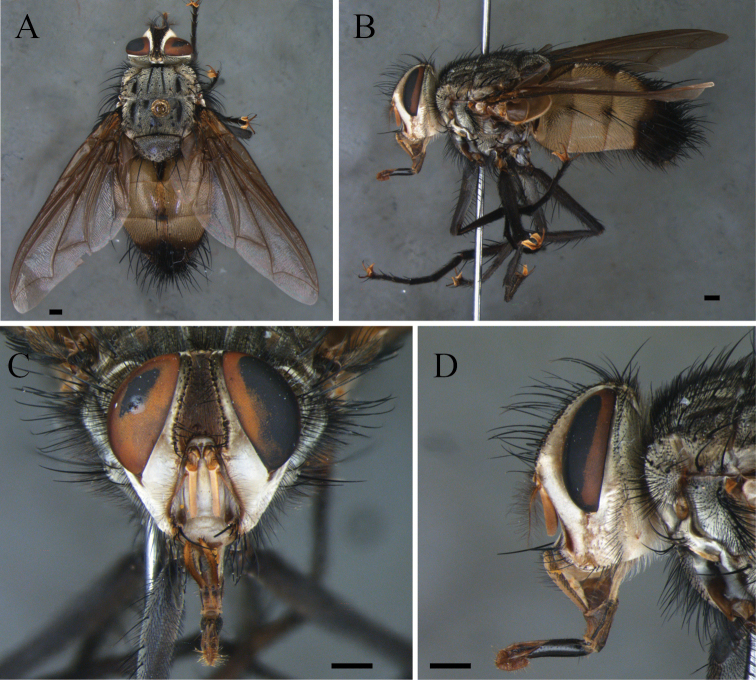
*Zelia magna* sp. nov., holotype, male. **A** Dorsal habitus **B** lateral habitus **C** head frontal **D** head lateral. Scale bars: 1 mm.

***Head***: Frontal vitta at its widest point ca. 1.2× as wide as the vertex in dorsal view. Frontal vitta, in the narrowest point, equal to bigger width than ocellar triangle. Fronto-orbital plate with 20 pairs of proclinate setae; broader than frontal-vitta and parafacial. Width of parafacial measured between inner margin of compound eye and antennal insertion is 2× the height of gena. Postpedicel slender, 2× the combined length of scape and pedicel; arista plumose with two to three dorsal and one ventral rows; length of longest cilia ca. 7× basal width of arista. Facial ridge with 16–14 setulae on lower third. Lower facial margin protruding, and visible in profile. Vibrissa long, inserted above lower facial margin. Prementum shorter than palpus. Labella developed, ca. 0.4× the prementum.

***Thorax***: Acrostichals 4+3. Dorsocentral 4+4. Intra-alar 2+3, first post-sutural weak; intra-postalar present. Supra-alar 2+3. Postpronotal lobe with five setae, four forming an anterior row and one posterior. Anepisternum with nine strong setae and two upward directed setulae anteriorly. Scutellum with one basal, one lateral, one weak subapical, one apical and two discal pairs of setae.

***Wing***: Base of R dorsally and ventrally setulose. M vein bent forward to R_4+5_, forming an angle bigger than 90°, and convex after bend.

***Legs***: Fore coxa with many setae anteriorly; fore femur with dorsal and posteroventral rows of setae; fore tibia with two posterior setae and row of shorter anterodorsal setae. Mid femur with three posteroventral basal setae, three dorsal to posterodorsal preapical setae; mid tibia with one anteroventral median seta, two anterior median setae and two posterior median setae. Hind femur with three anteroventral setae on basal half and three ventral setae on basal half and with row of anterodorsal setae; one posterodorsal preapical seta; hind tibia with two anterior median, two anteroventral median and two posterodorsal median setae. Claws straight with the tip curved.

***Abdomen***: Syntergite I+II without pair of median margin setae. Tergite III with three discal setae increasing in size anteriorly, one median marginal seta and two lateral marginal setae. Tergite IV with four discal setae increasing in size anteriorly and a marginal row of setae.

***Terminalia*** (Figs 7 D–F): Tergite VI brown and segment VII+VIII brown to tawny with silver pruinosity. Surstyili in lateral view wide, more than 2× the maximum cerci width; triangular shaped, slightly rounded at the apex. Surstyli in posterior view with small expansion laterally. Apex of the hypandrium directed backwards. Middle bar slightly shorter than total length of the granular structure of distiphallus. Apex of distiphallus curved.

#### Type locality.

Brazil, Rio de Janeiro, Nova Friburgo, Mury.

#### Distribution.

Brazil (Rio de Janeiro and São Paulo states).

#### Etymology.

The name refers to the size of this species, being the biggest of the species group. “Magna” (Latin) = large.

### Zelia guimaraesi

Taxon classificationAnimaliaDipteraTachinidae

Dios & de Santis sp. nov.

68173D52-1C0C-5FA3-8696-9AD407495F60

http://zoobank.org/A0CF312A-A8CC-4549-A05F-C955CD868ED1

**Figure 3.**
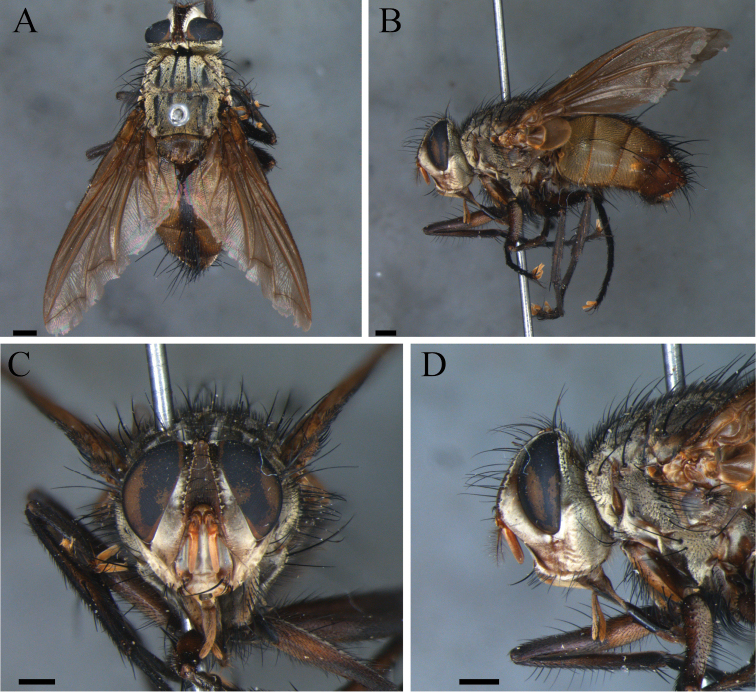
*Zelia guimaraesi* sp. nov., holotype, male. **A** Dorsal habitus **B** lateral habitus **C** head frontal **D** head lateral. Scale bars; 1 mm.

**Figure 4.**
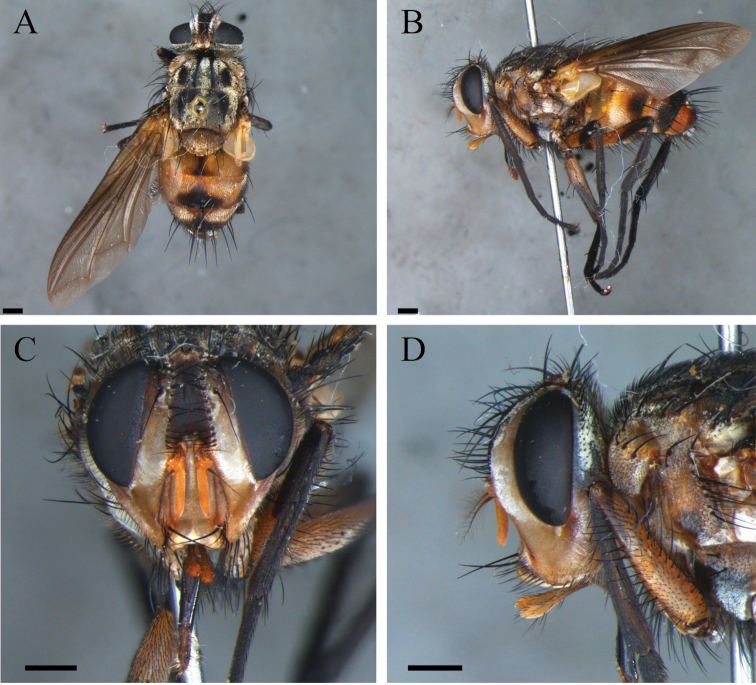
*Zelia guimaraesi* sp. nov., paratype, female. **A** Dorsal habitus **B** lateral habitus **C** head f rontal **D** head lateral. Scale bars: 1 mm.

Figs 3 , 4 , 7 G–I , 8

#### Type material examined.

***Holotype*** ♂: Brazil: *Santa Catarina*, Nova Teutônia, ii.1966, F. Plaumann col. (MZSP). Labelled as follows: “Brasilien / Nova Teutônia / 27°11B 52°23L, 300–500 m / ii.1966 / Fritz Plaumann” “Zelia / guimaraesi sp. nov. / Dios & Santis det. 2016” [handwriting/printed label]; “Holotipo” [red label].

***Paratypes***: Brazil: *Rio de Janeiro*, Nova Friburgo, “Mury, Nova Friburgo / Rio de Janeiro – Br. / 12.xi.1970 / Gred & Guimarães col.” 1 ♂ (MZSP); Petrópolis, Le Vallon, Alt, Mosella, 1 ♀, 1.ii–8–iii.1957, Albuquerque col. (MNRJ); São Paulo, Salesópolis, (Est. Biol. Boracéia / Salesópolis, SP-Br / 22 – 24.x.1982 / EXC. MZUSP col.) 1 ♂, (MZSP); Santa Catarina, Nova Teutônia, 1 ♂, same as holotype, 1 ♀, iv.1967, 1 ♀, xi.1966, 4 ♂, ix.1967, F. Plaumann col. (MZSP); *Rio Grande do Sul*, Santo Augusto, 1 ♂, i–ii, 1962, Roppa col. (MNRJ).

#### Diagnosis.

Parafacial larger or equal to 1/3 of the head width; tergite V entirely reddish brown; facial ridge with only one to two setulae; halter stem dark yellow; wings smokier alongside the veins; frontal vitta larger.

#### Description.

***Body length***: 12.0 mm.

***Coloration***: Frontal vitta and ocellar triangle dark brown to black. Head light yellow to tawny covered entirely with silver pruinosity. Occiput with long and black setulae. Postpedicel orange, but distal ¼ brownish orange. Palpus yellow-tawny. Thorax brown to dark brown with silver or light golden pruinosity; scutum with four dark vittae, in prescutum the two inner vittae are thinner than the outer, in postscutum, the inner vittae half the length of the outer, neither reaching the scutellum. Scutellum dark brown, with pale pruinosity posteriorly. Subscutellum with pale pruinosity. Wing hyaline, slightly light brown along the veins. Calypteres slightly infuscated. Halter yellowish to brownish. Posterior spiracle light brown. Legs brown to tawny with silver pruinosity on coxae and femora; tarsi darker. Claws brown, pulvilum yellow. Abdomen pale yellow, with median brown longitudinal vitta covering syntergite I+II, continuing along the middle of the abdomen to the end of tergite IV; tergite V entirely reddish black.

***Head***: Frontal vitta at its widest point ca. 1.1× as wide as the vertex in dorsal view. Frontal vitta, in the narrowest point, equal to bigger width than ocellar triangle. Fronto-orbital plate with 12–13 pairs of proclinate setae; narrower than frontal vitta and parafacial. Postocellar proclinate. Orbital plate with six setulae. Width of parafacial measured between inner margin of compound eye and antennal insertion is 2× the width of gena. Postpedicel slender, 1.5× the combined length of scape and pedicel; arista plumose with two to three dorsal and one ventral rows; length of the ventral cilia longer than the dorsal, longest cilia ca. 7× basal width of arista. Facial ridge with two to three setulae on lower third. Lower facial margin not protruding, and invisible in profile. Vibrissa long, inserted above lower facial margin. Premuntum as long as palpus. Labella developed, little longer than 0.5× the prementum.

***Thorax***: Acrostichals 2–3+2. Dorsocentral 3+3–4. Intra-alar 1+2–3, first post-sutural weak; intra-postalar absent. Supra-alar 2+2–3, first post-sutural weak. Postpronotal lobe with four setae, three forming an anterior row and one posterior. Anepisternum with eight strong setae and with one to two upward directed setulae anteriorly. Scutellum with one basal, one apical and one discal pairs of setae.

***Wing***: Base of R dorsally and ventrally setulose. M vein bent forward to R_4+5_, forming an angle slightly smaller than 90°, and convex after bend.

***Legs***: Fore coxa with many setae anteriorly; fore femur with dorsal and posteroventral rows of setae; fore tibia with two posterodorsal setae and row of shorter anterodorsal setae. Mid femur with three anteroventral on apical third, tibia with two posterodorsal, two anterodorsal and one anteroventral setae, two dorsal, one ventral, one posteroventral and one anteroventral preapical setae; mid tibia with one anteroventral median seta, two anterior median setae and two posterior median setae. Hind femur with three anteroventral setae on basal half and three ventral setae on basal half and with row of anterodorsal setae; one posterodorsal preapical seta; hind tibia with two anterior median, two anteroventral median and two posterodorsal median setae. Claws straight with the tip curved, same length as 5^th^ tarsomere.

***Abdomen***: Syntergite I+II without pair of median margin setae. Tergite III with two or none discal setae increasing in size anteriorly, one median marginal seta and one lateral marginal seta. Tergite IV with four discal setae increasing in size anteriorly and a marginal row of setae.

***Terminalia*** (Fig. 7 G–I): Tergite VI and segment VII+VIII yellowish tawny with scarce silver pruinosity. Surstyili in lateral view wider, more than 2× the maximum cerci width; triangular shaped, slightly narrowed at the apex. Surstyli in posterior view with an expansion laterally. Apex of the hypandrium directed upwards. Middle bar longer than the total length of the granular structure of distiphallus. Apex of distiphallus curved.

***Female differs from male by the following*** (Fig. 4): Body length, 12.4 mm. Fronto-orbital plate with two reclinate orbital setae, and one proclinate orbital setae. Inner vertical and outer vertical setae well developed and reclinate. Palpus swollen apically. Postscutum with two black vittae laterally on anterior half. Mid and hind legs yellow, but posteroventrally ¼ brownish black. Pulvillus and claws not elongated. Abdomen yellowish orange, with tergite III with a triangular spot starting posteriorly, and laterally with a brownish black spot at the insertion of lateral margin setae, tergite IV posteriorly with a horizontal brownish black band, laterally and medially almost reaching anterior margin, and tergite V entirely yellowish orange. Tergite III-V with an anteriorly band of pruinosity, in all the tergite.

***Terminalia*** (Fig. 8): Tergite VI and VII complete dorsally, with setae in all tergite VI and with few setae on the posterior margin of tergite VII, 6^th^ spiracle on membrane ventrally, before tergite VI and 7^th^ spiracle ventrally between tergite VI and sternite VIII. Tergite VIII present as thin and incomplete structure, with its apex elongated dorsally; very close to sternite X ventrally. Sternite VI and VII complete ventrally, with few setae in all posterior margin. Sternite VIII subrectangular as a narrow strip with setulae in the entire surface. Sternite X, as a narrow strip, with setula only on the posterior margin. Cerci well developed, sub-circular, with several setae apically with lingulae. Syntergite IX+X absent. Three spermatheca; equal sized; oval with apical portion with a pore and surface entirely rugose.

#### Type locality.

Brazil, Santa Catarina, Nova Teutônia.

#### Distribution.

Brazil (Rio de Janeiro, São Paulo and Santa Catarina states).

#### Etymology.

*Z. guimaraesi* sp. nov. is named in honor of the late Dr. José Henrique Guimarães, the former curator of Diptera at the MZSP, who contributed greatly to the study of Neotropical Tachinidae.

### Zelia formosa

Taxon classificationAnimaliaDipteraTachinidae

Dios & de Santis sp. nov.

53099F28-9A8E-5D34-87CA-99AD67E5376E

http://zoobank.org/102D8C26-F10C-4852-BBC1-AD8C88E13D8F

Figs 5 , 6

#### Type material examined.

***Holotype*** ♂: Brazil: *Santa Catarina*, Nova Teutônia, 1.ii.1961, F. Plaumann col. (MZSP). Labelled as follows: “Brasilien / Nova Teutônia / 27°11B 52°23L, 300–500 m / 1.ii.1961 / Fritz Plaumann” «Zelia / formosa sp. nov. / Dios & Santis det. 2016» [handwriting/printed label]; «Holotipo» [red label].

***Paratype***: Brazil: *Santa Catarina*, Nova Teutônia, 1 ♂, i.1940, 1 ♀, 14.v.1963, F. Plaumann col. (MZSP).

#### Diagnosis.

Postpedicel almost entirely brownish black, but 1/6 ventrally yellowish tawny; fronto-orbital plate entirely silvery pruinose; wing with a maculae in its base; R_4+5_ dorsally setulose for 1/4 of distance to cross vein dm-cu; postscutum, with pale pruinosity only on the anterior region, not forming vitta; syntergite I+II, with one pair of median marginal setae; abdomen with syntergite I+II to tergite IV pale yellow, with a brownish black vitta on middle, broadening posteriorly (three times the length of the anterior portion) and with white pruinosity on the lateral of each tergite.

#### Description.

***Body length***: 12.4 mm.

***Coloration***: Occiput with pale setulae. Postpedicel light brown, but proximal ¼ orange. Palpus yellow-orange. Postscutum, with pale pruinosity only on the anterior region, not forming vitta. Scutellum dark brown, with pale pruinosity posteriorly. Subscutellum with pale pruinosity. Wing hyaline, slightly light brown along the veins. Calypteres white-pale translucent. Halter and posterior spiracle light brown. Legs brown with silver pruinosity on coxae and femora; tarsi darker. Claws brown with tip darker. Abdomen pale yellow, with median brown longitudinal vitta covering syntergite I+II, in tergite III a brownish black dot at the insertion of the median marginals, in tergite IV a brownish black triangular spot on the posterior region and in V; and tergite V entirely brownish black without pruinosity; posterolateral margin of tergite IV with a brown spot.

**Figure 5.**
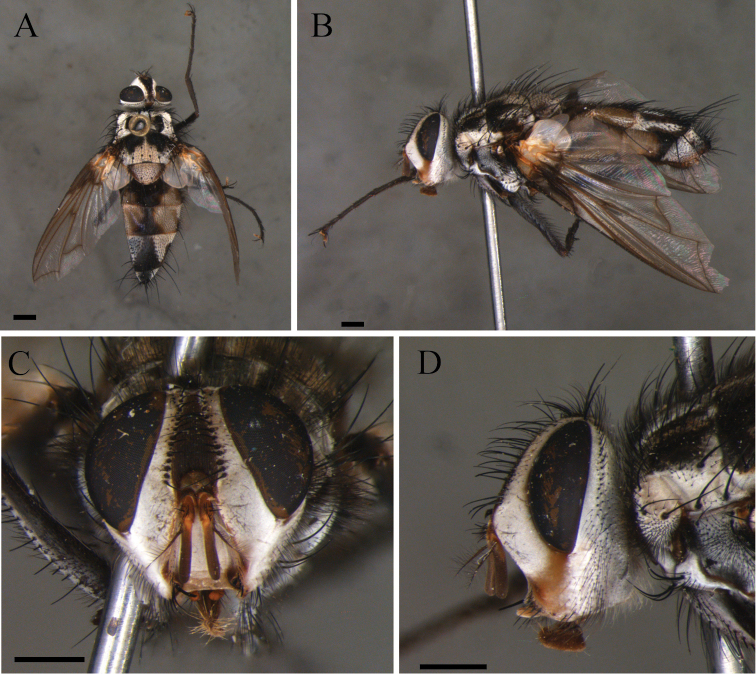
*Zelia formosa* sp. nov., holotype, male. **A** Dorsal habitus **B** lateral habitus **C** head frontal **D** head lateral. Scale bars: 1 mm.

***Head***: Frontal vitta at its widest point ca. 1.2× as wide as the vertex in dorsal view. Frontal vitta, in the narrowest point, equal to width than ocellar triangle. Fronto-orbital plate with 20 pairs of proclinate setae; broader than frontal-vitta and parafacial. Width of parafacial measured between inner margin of compound eye and antennal insertion is 2.5× the height of gena. Postpedicel slender, 2.2× the combined length of scape and pedicel; arista plumose with two or three dorsal and one ventral rows; length of longest cilia ca. 7× basal width of arista. Facial ridge with 14–16 setulae on lower third. Lower facial margin not protruding, and invisible in profile. Vibrissa long, inserted above lower facial margin. Premuntum shorter than palpus. Labella developed, ca. 0.4× the prementum.

***Thorax***: Acrostichals 4+3. Dorsocentral 4+4. Intra-alar 2+3, first post-sutural weak; intra-postalar present. Supra-alar 2+3. Postpronotal lobe with five setae, four forming an anterior row and one posteriorly. Anepisternum with nine strong setae and two upwardly directed setulae anteriorly. Scutellum with one basal, one lateral, one weak subapical, one apical, and two discal pairs of setae.

***Wing***: Base of R dorsally and ventrally setulose. M vein bent forward to R_4+5_, forming an angle slightly smaller than 90°, and convex after bend.

***Legs***: Fore coxa with many setae anteriorly; fore femur with dorsal and posteroventral rows of setae; fore tibia with one posterior seta and a row of shorter anterodorsal setae. Mid femur with two anteroventral, three posteroventral basal setae, three dorsal to posterodorsal preapical setae; mid tibia with one anteroventral median seta, two anterior median setae, one posteroventral median seta and four preapicals setae. Hind femur with three anteroventral setae on basal half and three ventral setae on basal half and with row of anterodorsal setae; one posterodorsal preapical setae; hind tibia with one anterior median, two very long anteroventral median and two posterodorsal median setae and one anteroventral and one posteroventral preapical setae. Claws straight with the tip curved.

***Abdomen***: Syntergites I+II with one pair of median marginal setae. Tergite III with three discal setae increasing in size anteriorly, one median marginal seta and two lateral marginal setae. Tergite IV with four discal setae increasing in size anteriorly and a marginal row of setae.

***Terminalia***: Tergite VI and segment VII+VIII yellowish tawny with silver pruinosity. The remaining of the terminalia of the only dissected male was lost in the preparation and cannot be described here.

**Figure 6.**
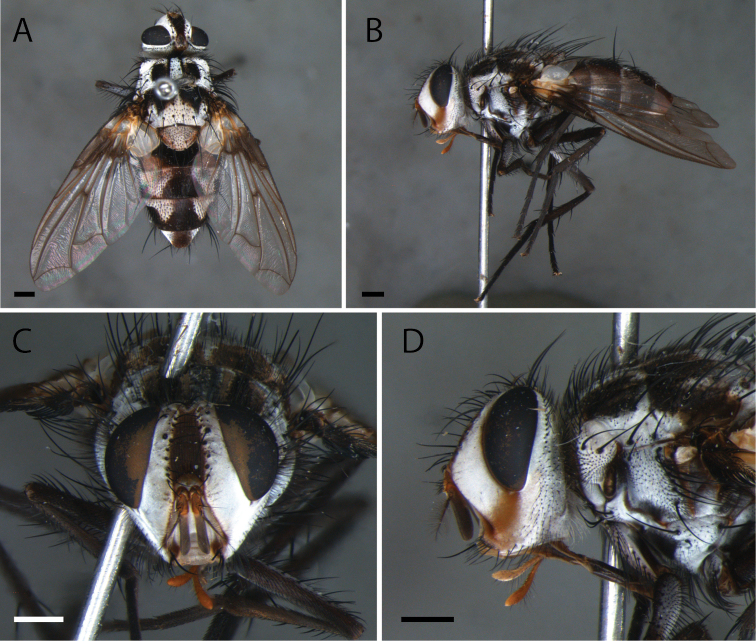
*Zelia formosa* sp. nov., paratype, female. **A** Dorsal habitus **B** lateral habitus **C** head frontal **D** head lateral. Scale bars: 1 mm.

***Female differs from male by the following*** (Fig. 6): Body length, 11.8 mm. Fronto-orbital plate with two reclinate orbital setae, and one proclinate orbital seta. Ocellar setae well developed and decussate. Palpus slightly more robust than in male. Pulvillus and claws not elongated. Abdomen oval. Tergites testaceous laterally white pruinose on syntergite I+II to tergite V; tergite V with row of marginals.

**Figure 7.**
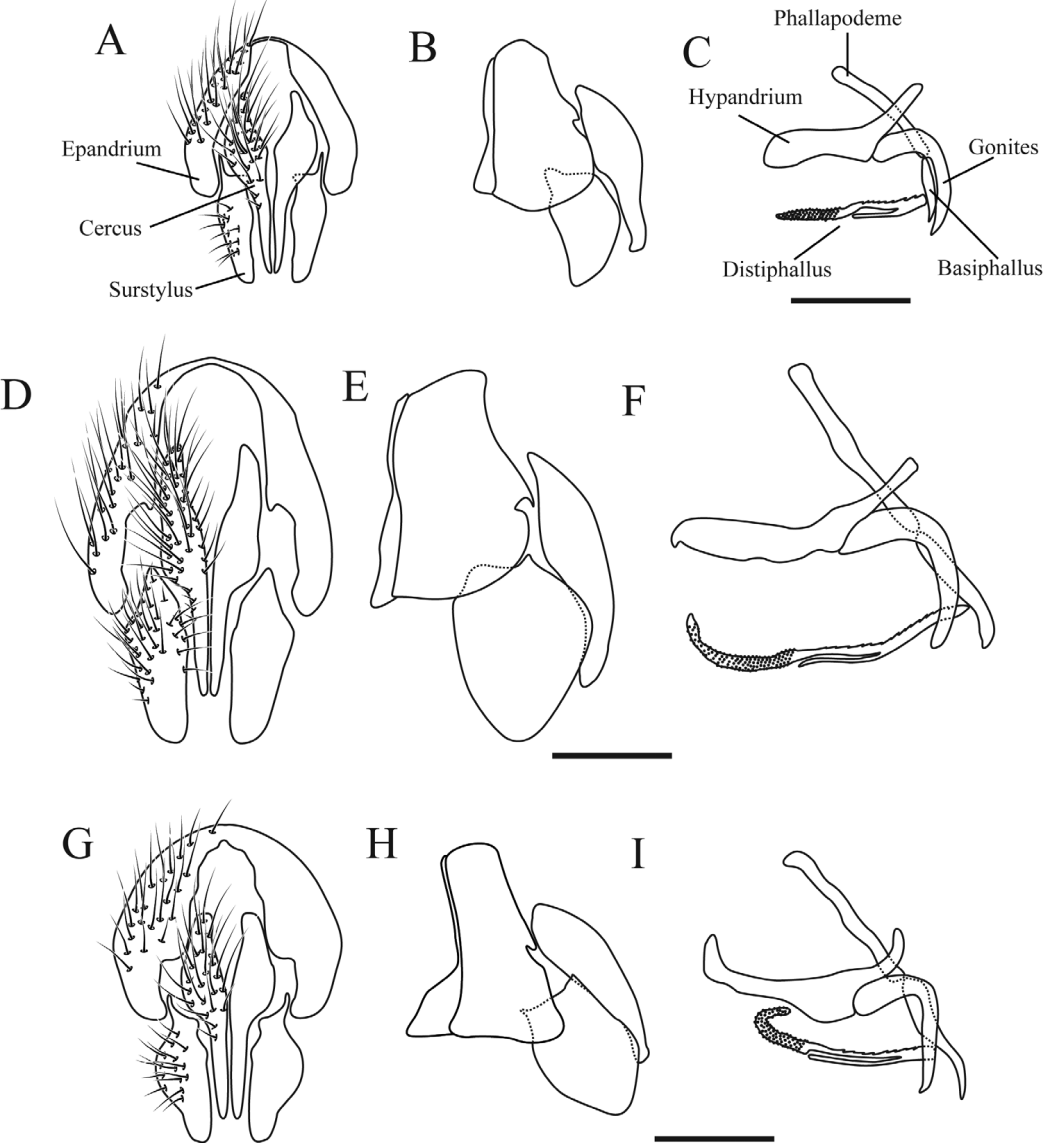
Male terminalia. **A–C***Zelia discalis***A** epandrium, cerci and surstylus in posterior view **B** epandrium, cerci and surstylus in lateral view **C** hypandrium, phallapodeme, gonites and aedeagus in lateral view. **D–F**Zelia magna sp. nov. **D** epandrium, cerci and surstylus in posterior view **E** epandrium, cerci and surstylus in lateral view **F** hypandrium, phallapodeme, gonites and aedeagus in lateral view. **G–I***Zelia guimaraesi* sp. nov. **G** epandrium, cerci and surstylus in posterior view **H** epandrium, cerci and surstylus in lateral view **I** hypandrium, phallapodeme, gonites and aedeagus in lateral view. Scale bars: 0.5 mm.

**Figure 8.**
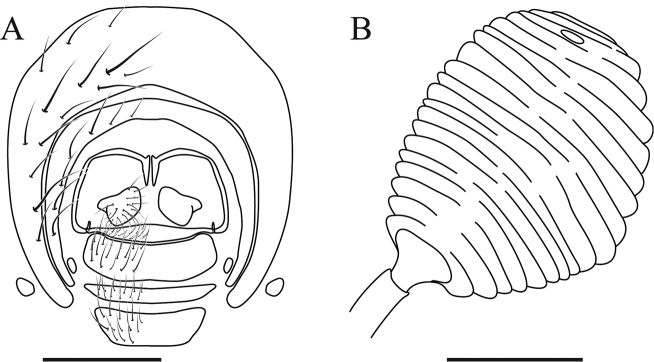
*Zelia guimaraesi* sp. nov. female terminalia. **A** Female terminalia in posterior view **B** spermatheca (only one represented, but three identical present). Scale bars: 0.5 mm (**A**), 0.1 mm (**B**).

#### Type locality.

Brazil, Santa Catarina, Nova Teutônia.

#### Distribution.

Brazil (Santa Catarina state).

#### Etymology.

The name refers to the unique and abundant pruinosity in the thorax and abdomen. “Formosa” (Latin) = beautiful.

## Supplementary Material

XML Treatment for Zelia

XML Treatment for Zelia discalis

XML Treatment for Zelia magna

XML Treatment for Zelia guimaraesi

XML Treatment for Zelia formosa
